# Conventional Microscopic Stapedotomy: An Obsolete Technique or Still the Gold Standard for the Management of Otosclerosis?

**DOI:** 10.7759/cureus.14126

**Published:** 2021-03-26

**Authors:** Alexandros Poutoglidis, Nikolaos Tsetsos, Chrysa Vardaxi, Georgios Fyrmpas, Frideriki Poutoglidou, Adamantios Kilmpasanis, Konstantinos Vlachtsis

**Affiliations:** 1 Department of Otorhinolaryngology-Head and Neck Surgery, “G. Papanikolaou” General Hospital, Thessaloniki, GRC; 2 Department of Clinical Pharmacology, School of Health Sciences, Aristotle University of Thessaloniki, Thessaloniki, GRC

**Keywords:** stapedotomy, otosclerosis, conductive hearing loss, otosurgery

## Abstract

Background and objectives

Clinical otosclerosis is a relatively common entity, accounting for 0.5%-2% of the general population. Otosclerosis is characterized by an abnormal bone formation in the temporal bone that eventually causes conductive hearing loss. Bilateral involvement is fairly common. Treatment can be either conservative with medications and a hearing aid, or surgical. Stapedotomy is considered, nowadays, the most effective surgical technique for the management of otosclerosis. The purpose of the present study is to present our long-term results with stapedectomy, the audiological outcome, as well as the complications encountered.

Subjects and methods

This is a retrospective single-centre study. All patients diagnosed with otosclerosis and treated operatively with a stapedotomy from January 2010 to December 2019 were included in the study. Demographic data, air and bone conduction thresholds, complications and length of the prosthesis were recorded.

Results

The study included a total of 72 patients. The audiological results showed a statistically significant improvement in the air conduction thresholds in all the affected frequencies (p<0.001). Post-operative complications included deterioration or severe hearing loss up to 100 dB (n=1, 1.39%), loss or distortion of taste (n=4, 5.6%) and tinnitus (n=2, 2.8%).

Conclusions

Our results demonstrate that stapedotomy is an effective technique for the management of otosclerosis. Stapedotomy, when performed by an experienced surgeon, provides excellent outcomes, with limited complications.

## Introduction

Otosclerosis is a progressive disease characterized by abnormal bony remodeling, which includes the formation of new bone and bone resorption in the temporal bone [1,2]. In most cases, otosclerosis is presented with conductive hearing loss (CHL) in the low frequencies with a Carhart notch [3]. However, progression of the disease in the cochlea may cause Sensorineural Hearing Loss (SHL) ‘‘hyalinization’’ of the spiral ligament and atrophy of the stria vascularis [4]. 

Surgical treatment has been evolved through the years. Restoration of hearing in CHL cases and improvement of patients’ quality of life are the main goals of treatment. Stapedotomy is considered the most effective, up-to-date, surgical technique for the management of otosclerosis. On the other hand, fenestration of the footplate can be performed (microscopically or endoscopically) either with the use of a micro-drill or a laser beam [5-7].

The aim of the present study is to present our long-term results with stapedotomy for the management of otosclerosis and compare them with the ones obtained with other techniques reported in the literature. Also, audiological outcomes and complication rates will be presented.

## Materials and methods

Data acquisition

The present study is a retrospective review of all patients treated with a stapedotomy for otosclerosis in our Otorhinolaryngology department from January 2010 to December 2019. The study was approved by the Human Research Ethics Committee of our Ηospital (approval number:0105012021), and it was conducted in accordance with the Declaration of Helsinki (1975) and its later amendments. A written informed consent was obtained by all patients. Patients with incomplete charts and patients with a follow-up of less than a year were excluded. A data extraction form was designed to record the following data: demographic characteristics (including age, gender and side), surgical time, length of prosthesis and complications.

All patients had undergone preoperative and postoperative pure tone audiograms of both air and bone conduction at the frequencies of 250 Hz, 500 Hz, 1 kHz, 2 kHz, and 4 kHz. The preoperative and the postoperative values of the air and bone conduction pure tone thresholds were compared.

Surgical technique

**Figure 1:**
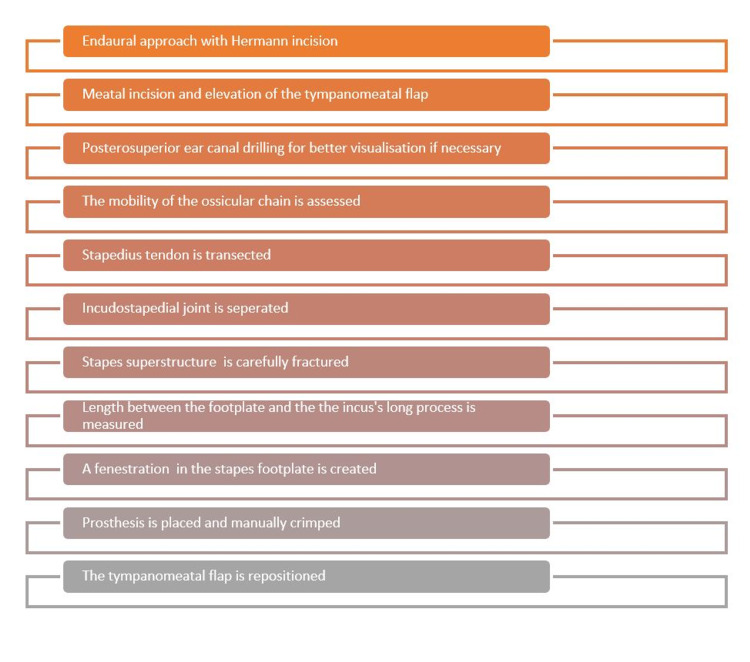
Surgical steps of the stapedotomy technique used in our Department.

Operations were performed by two experienced surgeons under general anesthesia. Briefly, in all the patients an endaural approach with Hermann incision was made, followed by meatal incision and elevation of the tympanomeatal flap. The posterosuperior part of the ear canal was drilled for better visualisation if necessary. The ossicular chain fixation was confirmed. Next, the stapedius tendon was transected and the incudostapedial joint was separated. The stapes superstructure was carefully fractured and the distance between the oval window and the long process of the incus was measured and an appropriate-sized prosthesis was selected. A fenestration 0.2 mm wider than the prosthesis was made in the posterior one-third footplate of the stapes, using the special needle (small fenestra). The Richards type platinum fluoroplastic prosthesis (0,6 mm shaft diameter) was then placed and manually crimped. The length of prosthesis was either 4.50 mm or 4.75 mm. The tympanomeatal flap was then repositioned to its normal position (Figure 1).

Statistics

Data were initially entered into an Excel sheet and were analyzed using the Statistical Program for Social Sciences (SPSS) 21.0 (IBM Corporation, Armonk, NY). Categorical variables were expressed as frequencies and percentages. The Shapiro-Wilk test was used to determine whether data were normally distributed. Our data were related to each other in a non-Gaussian distribution; we, therefore, used Wilcoxon signed-rank test for intragroup testing. A p-value of less than 0.05 was considered statistically significant.

## Results

The study included a total of 72 patients, 25 males (35%) and 47 females (65%). The mean age was 44 years (median 45). The length of prosthesis was either 4.5 mm (n=47, 65%) or 4.75 mm (n=25, 35%).The mean (± SD) follow-up period was 13.6 (± 1.2) months. Postoperative deterioration or severe hearing loss up to 100 dB was encountered in one patient (1.39%), loss or distortion of taste in four patients (5.6%) and tinnitus in two patients (2.8%). Mean time of stapedotomy was 35 minutes and no other significant complications were recorded.

**Figure 2:**
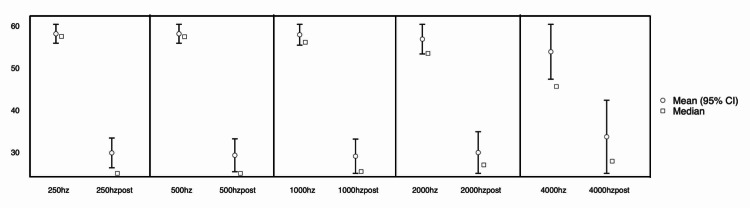
Audiometric improvement after stapedotomy (250-4000 Hz).

The audiological results showed a statistically significant improvement in the air conduction thresholds in all tested frequencies (250 Hz, 500 Hz, 1000 Hz, 2000 Hz, 4000 Hz) (p<0.001). The mean difference in the air conduction thresholds preoperatively and postoperatively was 30 dB in 250 Hz and 500 Hz, 27.5 dB in 1000 Hz, 22.5 dB in 2000 Hz and 15 dB in 4000 Hz. The differences in the air conduction after the stapedotomy are presented in Table 1 and Figure 2. Bone conduction thresholds remained almost unchanged after surgery and air-bone gap closed to <10db in 95% of cases. Finally, the length of prosthesis did not influence the audiological outcomes.

**Table 1:** Pre- and post-operative air conduction thresholds.

Hz	Mean difference (dB)	p	CI
250	30	<0.001	27.5-325
500	30	<0.001	27.5-32.5
1000	27.5	<0.001	25-30
2000	22.5	<0.001	20-25
4000	15	<0.001	10-17.5

## Discussion

Clinical otosclerosis is a relatively common disease and its incidence is estimated to be between 0.5% and 2% [8]. According to the literature, otosclerosis is characterized by a female predominance, with a female/male ratio of 2/1, and a median age of onset of 47 years (range 20-75) [9-11]. Our demographic data are similar to those reported in the literature.

Micro-drills and laser beams are often employed to increase the precision in the fenestration of the stapes footplate and to decrease the possibility of footplate fracture or mobilization. It has been suggested that modern equipment reduces the postoperative complications and improves the final audiological outcomes [12]. However, other studies demonstrate that the audiological outcomes in the laser and in the conventional stapedotomy are similar [6]. The CO2 laser has been associated with thermal effects on the inner ear perilymph. Acoustic trauma or even penetration of the neuro-endothelium by Argon and KTP Lasers has been a concern about their use [13-16]. Moreover, there have been cases of delayed facial palsy related to KTP lasers [17]. However, no statistically significant difference between laser and non-laser stapedotomy regarding complications such as sensorineural hearing loss and tinnitus and vertigo was shown in a meta-analysis [18]. As for our series, only two patients (2,7%) suffered a severe, persistent tinnitus. 

It is important to highlight the role of preoperative CT scan imaging. CT scan is not only recommended for diagnostic purposes but mainly to exhibit possible surgical risks [19]. In our department, nine patients were treated conservatively (bisphosphonates or a hearing aid) because of anatomical abnormalities in the CT scan that could have potentially led to severe postoperative complications, like facial nerve injury or SHL. It is worth mentioning that most studies in the literature assessing the outcomes of conventional stapedotomy do not involve the use of CT scan imaging. Thus, some complications were not due to the lack of modern equipment but due to the lack of pre-operative imaging. Several imaging details need to be evaluated, including the facial nerve course, the concurrent middle and inner ear pathology, the status of the oval and round window, the ossicular chain integrity and the jugular bulb location [20,21]. Otosclerosis is a disease that, in most of the cases, affects the hearing bilaterally [22]. Therefore, we suggest limited or no interventions in cases of anomalies that pose the risk of causing SHL.

The cost of stapes surgery varies and primarily depends on the equipment that is selected. The application of lasers in stapes surgery and especially the CO2 laser has been significantly linked to increased cost [23].

## Conclusions

The present study indicates that conventional stapedotomy is a safe procedure providing good audiological outcomes and at the same time remains cost-efficient. Preoperative CT scan imaging is mandatory to avoid intraoperative and postoperative complications. There is not yet evidence to prove that laser fenestration of the footplate is superior to the conventional technique and further prospective studies to investigate this aspect are needed.
